# Registering transparency: the making of the international clinical trial registry platform by the world health organization (2004–2006)

**DOI:** 10.1186/s12992-023-00970-5

**Published:** 2023-09-18

**Authors:** Loreto Fernández-González

**Affiliations:** 1 Dalla Lana School of Public Health, University of Toronto, 155 College St, Toronto, ON Canada; 2 Clinical and Epidemiological Research Unit, Instituto Oncológico Fundación Arturo López Pérez, José Manuel Infante 805, Santiago, Chile

**Keywords:** Pharmaceutical Research, World Health Organization, Governance, Clinical trial

## Abstract

**Background:**

This paper examines the events and conditions that led to the creation of the International Clinical Trials Registry Platform (ICTRP) in 2006 by the World Health Organization (WHO), and how the WHO addressed the issue of transparency in global pharmaceutical research. Using historical textual analysis, I trace the scientific debates that advocated for the establishment of official clinical trial registries in medical journals, and the sequence of actions following the GSK Paxil scandal in 2004, identifying the major ethical and scientific arguments that led to the involvement of the WHO as a key actor in trial registration in the context of the Big Pharma business model.

**Results:**

Through the questions “Why register?” and “Why registries?” as a roadmap, I examine the issues of publication bias and selective reporting by the industry, scrutinizing two ways in which the practice of publication bias damaged transparency in industry-sponsored research. The first involved ethical concerns regarding human subject exploitation and concealing of negative results. The second addresses the deterioration of the certainty of evidence due to incomplete access to trials results. By reviewing the series of events that occurred between 2004 and 2006 –between the Paxil scandal and the launch of the ICTRP—, I analyze the actions taken by the different actors involved: (1) the International Committee of Medical Journal Editors (ICMJE) and the creation of the Ottawa Group; (2) the WHO, beginning with the Ministerial Summit on Health Research held in November of 2004, and (3) the responses of the pharmaceutical industry and specifically GSK to the call for transparency and trial registration.

**Conclusions:**

The history of trial registration through the ICTRP as a dataveillance apparatus shows the difficulty of regulating a health enterprise turned into a global business. Moreover, it shows the challenges of globalization and how easier and faster it is to globalize business compared to good practices, raising the question of why it has been so hard to undo these trends. Indeed, the history of the movement for trial registration is not a history of regulation success, or at least not yet.

## Background

When on June 2nd of 2004, the *New York Times* informed about the lawsuit against GlaxoSmithKline (GSK) by the New York Attorney General Eliot Spitzer at the NY State Supreme Court, the harm of non-disclosure of results of clinical trials became a public controversy in the United States. In the lawsuit, Spitzer argued that the pharmaceutical company had committed fraud “by withholding negative information and misrepresenting data on prescribing its antidepressant Paxil to children” [1], referring to the increased risk of suicide in children taking Paxil (paroxetine) compared to placebo in a clinical trial [2]. The study in question corresponds to “Study 329”, which was conducted between 1994 and 1998 in the US and Canada. Although it was eventually published in 2001 [3], in 1998 an internal memo circulated in the company advising against the disclosure of negative results, including increased suicidal risk compared to placebo: “It would be commercially unacceptable to include a statement that efficacy had not been demonstrated, as this would undermine the profile of paroxetine.” [4]. Moreover, the published article by the Journal of the American Academy of Child and Adolescent Psychiatry (JAACAP), although it included data about suicidal risk in one table, concluded that it was well tolerated and effective in adolescents. Even though the matter of selective reporting and publication of evidence had been object of scientific debate for decades, and this case of research malpractice was not the first to be documented, it constituted a decisive event for the involvement of the World Health Organization (WHO) in the issue of transparency regarding clinical trial registration and disclosure of results by the pharmaceutical industry.

Indeed, the case against GSK’s Paxil —which came to be nicknamed as “suicide pills” by the press— triggered a series of responses from different key actors related to medical research and their regulation, as the lawsuit was later replicated in other countries [5]. An editorial by the medical journal The Lancet a few days after the lawsuit commented: “If GSK has nothing to hide, as it claims, it should open its files before being ordered to do so by a court—and do so right now.” [6]. One of the major issues raised in the debate was the acknowledgment by regulatory entities about the existence of the trials, both past and ongoing. Scandals like Paxil revealed that the tracking and registration of these trials was incomplete and scattered. The massive scale to which clinical trials were recruiting human participants made unanswerable the simple question of how many trials were being conducted at a specific moment. Moreover, the increasing implementation of clinical trials outside the US and Western Europe had made the task of holding the pharmaceutical companies accountable for reporting the trials conducted in low- and middle-income countries increasingly difficult. It was in this complex scenario, that the WHO decided to address the situation and become an actor in the debate for clinical trial registration, committed to promote transparency in clinical research.

The present paper aims to examine the events and conditions that led to the creation of the International Clinical Trials Registry Platform (ICTRP) in 2006 by the WHO, and how the WHO addressed the issue of transparency with pharmaceutical research. Tracing the scientific debates that advocated for the establishment of official clinical trial registries in medical journals and highlighting the sequence of actions following the GSK Paxil scandal in 2004, I aim to identify the major factors that led to the involvement of the WHO as a key actor in the promotion of trial registration, with the launch of the ICTRP in 2006. Although influential public clinical trial registries were already in force by the date it was created —the most relevant being the Food and Drug Administration platform www.clinicaltrials.gov by the US government—, the involvement of the WHO in the arena of clinical trial transparency and registration should be read in terms of power dynamics in the era of the industrialization of clinical research [7]. In other words, the WHO initiative cannot be fully understood without considering the exponential economic growth and expansion experienced by pharmaceutical companies in the last decades. It is in this expansion of the drug industry —where developing medications constitutes a business model—, that the call for transparency and accountability by the major global health agency must be contextualized.

## Methods

This article presents the results of a historical textual analysis research. Sources can be grouped into three main categories. First, the scientific literature published in peer-reviewed medical journals and books that make reference to the issues related to trial registration and concerns about scientific transparency and patient safety. Specifically, I trace how trial registration begins to be proposed in the medical literature as the main strategy to enforce transparency by the pharmaceutical industry, and the arguments given by experts in the topic. A second source of texts analyzed correspond to WHO official documents, mainly news releases in its dissemination platforms expressing the WHO standpoint on the matter, official resolutions from the 2005 World Health Assembly where trial registration is mentioned, and subsequent statements and standard sets published by the WHO. Finally, other sources belonging to key actors in the pharmaceutical sector in response to the WHO actions are quoted and discussed.

All sources were retrieved electronically from scientific journal databases, or official websites when available. Texts were closely read and analyzed looking for key terms and ideas related to the topic and the events narrated. In scientific journal articles, references were reviewed to ensure the inclusion of all relevant sources and interlocutors addressed, and search engines (Medline and Google Scholar) were utilized to track the upcoming citing articles and pieces published such as press releases and news articles that continued the debate on the matter. Navigation in the WHO archival database was performed with the support of an expert health sciences librarian from the University of Toronto in April of 2019. Search on databases did not follow a pre-established search strategy such as time frames and selected keywords, privileging an inductive and flexible approach guided by the emergence of new sources and connections between them.

A chronological timeline was developed to establish the key events and identify the main voices (either scientists or editorial boards, as we will see). This timeline was enriched through an iterative process of going back and forth through the sources in order to get a comprehensive portrayal of the issue, the terms utilized, and the actors addressed and/or interpellated. Central arguments and themes were extracted and analyzed as detailed below. Notably, the reader will notice that even though the critical events discussed in the article refer to the period between 2004 and 2006, the historical analysis begins with sources dated years, even decades, earlier. This was done in an effort to understand the context where the debate and events took place, particularly the political economy of clinical trials and the Big Pharma model of drug development, by the late twentieth century. In this sense, this article does not aim to merely enumerate a list of events and publications, but to trace the power dynamics that shaped the politics of trial registration, in an attempt to “tell a story” of this debate, of which these press releases, statements and scientific articles are the textual vestiges.

I begin by reviewing the recent history of the pharmaceutical industry and its state in the early 2000’s, both in terms of technological development and economic growth characterized by strong investment in Research & Development (R&D), as well as the establishment of the Big Pharma business model. I then delve into the scientific debate that took place in medical journals advocating for the necessity of trial registration systems. Engaging with the questions “Why register?” and “Why registries?” as a roadmap, I examine the issue of publication bias and selective reporting by the industry. I scrutinize two ways in which the practice of publication bias damaged transparency in industry-sponsored research. The first involved ethical concerns derived from cases of human subject exploitation and concealing of negative results, both in trials conducted in the US and in developing countries. The second concern has to do with the deterioration in the certainty of the evidence —especially meta-analysis and systematic reviews— due to incomplete access to trials results, leading to inaccurate conclusions about the true effectiveness of novel medications.

Next, I examine the series of events that occurred between 2004 and 2006, that is, between the Paxil scandal and the launch of the ICTRP in May of 2006. I will analyze the actions taken by the different actors involved: (1) the International Committee of Medical Journal Editors (ICMJE) and the creation of the Ottawa Group; (2) the WHO, beginning with the Ministerial Summit on Health Research held in November of 2004, and (3) the responses of the pharmaceutical industry and specifically GSK to the call for transparency and trial registration. Finally, I reflect on the impact and significance of the establishment of the ICTRP as a dataveillance apparatus in the context of neoliberal globalization, both by analyzing the formal endorsement by the Helsinki Declaration, and by providing some insight on the issue of compliance with trial registration in the upcoming decade after its inception.

## Results

### The industrialization of pharmaceutical research and the problem of transparency in the early 2000s

The history of the pharmaceutical industry during the past five decades has been characterized by a “biotechnology revolution” (between 1970s and the 2000s), and a ‘Winter of Discontent?’ (2000–2010) [8]. Strictly speaking, the events related to trial registration correspond to this last era, but the circumstances that led to the implementation of registries must be traced back to the so-called revolution.

In this sense, the biotechnology revolution era does not only refer to an increase in technological development, but also to a redefinition of the geopolitics of the pharmaceutical industry embedded in neoliberal macroeconomic processes. One of these main redefinitions consisted in the shift from Europe to the US in terms of R&D dominance due to changes in the patent regimes and the large investment and strengthening of public funding for health research in the early 1970s, including the National Research Act of 1974 [8], [9]. This period was also characterized in the US by the creation and implementation of ethical regulatory codes for clinical trials with human subjects which, along with FDA establishment of randomized controlled trials (RCT) as the gold standard for assessing safety and efficacy of new therapies by 1970, boosting clinical trials [10].

In 2000, Richard Rettig [7] described the scenario of clinical research as essentially industrialized: “a large, rapidly growing line of business.”, especially in the field of drug development. During this period —partly influenced by the new information technology industry— pharmaceutical companies underwent a process of strong commercialization of scientific research, venture capital, and Intellectual Property Rights (IPR) [8]. The growing demand for new drugs both in high and low- and middle-income countries created large markets, resulting in increasingly high revenues for companies [10]. Investing in R&D became a growing interest for the drug industry throughout the 1980s, displacing public funding by the National Institutes of Health (NIH) by 1992 [10]. The implementation of the “Big Pharma” model of business [11] also consisted in large scale practices of out-sourcing (primarily through Contract Research Organizations) and off-shoring to low- and middle-income countries [10].

However, this rapid financial growth and geopolitical expansion, wasn’t exempt of problems or crisis by the turn of the century. The “Winter of discontent?” that Malerba and Orsenigo refer to when describing the situation of the drug industry during the 2000s, is named so because of “growing doubts about the sustainability of the business model” [8], and a deterioration of public opinion due to scandals like Paxil and heated debates regarding access to healthcare and prices of medications worldwide. Indeed, by the early 2000s, the Big Pharma business model, dominant in the 1990s, was being highly criticized: “The blockbuster business model that underpinned Big Pharma’s success is now irreparably broken. (…) The pharmaceutical industry is a prisoner of its past successes.” [11].

This difficulty to adapt was not only regarding its financial and R&D model, but its role in society. By 2004, the industry was under public scrutiny for what was perceived as a generalized lack of transparency and setting priorities that prepended profit rather than patient safety and access. A growing literature condemning malpractices of political lobby, biased results of new drugs’ effectiveness, and generalized misleading marketing, appeared in scientific and non-scientific outlets, including a book by the former editor of the New England Journal of Medicine, Marcia Angell titled: “The truth about the Drug Companies: How they deceive us and what to do about it” [12].

This was the general background where the debate on trial registration took place. How could registering trials in a specific platform help with these “deceptions”, in Angell’s words? Rettig’s paper concludes with the following reflection:

“Transparency. This concern links all questions in the clinical research domain. Concerns have been raised about suppression of research results by drug firms, bias in interpreting inconclusive research as a function affiliation with nonprofit or for-profit institutions, multiple reporting of the results of a single trial, and ghost authorship of articles, especially among nonacademic investigators for whom publishing is not a strong incentive. Among the remedies suggested, *the most significant is the proposal to register all clinical trials at inception*, whether governmentally or privately sponsored.” (italics by me) [7].

The connection between transparency and registration was already well established. Nonetheless, it took years for the WHO to step into the debate.

### Why registries? why register?

Rettig’s quote shows that there was no single motive to advocate for the implementation of a trial registry. “Transparency concerns” is a broad notion that designates several issues happening in the drug industry at the time. For this reason, it is important to distinguish these problems and observe the arguments used to justify both the existence of a registry, as well as the mandatory registration of trials, as a “remedy” [7]. This distinction is also important to examine how the WHO addressed each of these issues and their arguments.

In one word, during the two decades prior to the launching of the ICTRP, the problem with transparency was framed as a problem of bias. “Not surprisingly, bias is now rampant in drug trials”, Angell wrote in 2004 [12]. In 1993, Kay Dickersin —one of the strongest advocates for registration and an expert in publication bias since the late 1980s— and Y. Min, defined: “Publication bias is any tendency on the parts of investigators or editors to fail to publish study results on the basis of the direction or strength of the study findings.” [13]. In other words, publication bias happens when the trial results do not go as expected, thus publishing the study does not favor one of the parties involved, and it remains unpublished. Bias may, however, also occur in the opposite way: overreporting positive results, and/or publishing them in different journals, “in slightly different forms” [12]. A related practice, selective reporting or misreporting, refers to publication of only part of the data: for example, the publication of subgroups of patients where the drug proved effectiveness (though not on the whole sample), and reporting specific time frames of the trial and not the outcomes of its whole duration according to the initial design [12]. By 2004, it was estimated that only half of existing trials were published [2], [14].

Although the study of publication bias and misreporting can be traced back to the 1950s, it was not until the 1960s that different registries specific to conditions or types of study became to be implemented in the US, according to Dickersin and Rennie [15]. The first mention of a trial registry gathering all areas of medicine can be found in a letter written by Thomas Chalmers in 1977 in the New England Journal of Medicine [16]. The first researcher to demonstrate the impact on publication bias regarding interpretation of clinical trial results was John Simes, in 1986 [17], when he compared the results of two different meta-analyses of chemotherapy in ovarian cancer and showing the divergent results depending on the studies included (only published vs. registered). More interestingly, in that same paper published in the Journal of Clinical Oncology, Simes called for the creation of an international registry of clinical trials, more than a decade before the FDA trial registry platform was launched, and two decades before the WHO’s platform.

Following Simes’ study, the upcoming years and 1990s decade had an active debate on the issue. Prominent figures in the discussion were Thomas C. Chalmers (who held positions at the National Institutes of Health), Sir Iain Chalmers (one of the founders of the Cochrane Collaboration), and Dickersin herself (who was mentored by both Thomas and Iain Chalmers in Boston during the 1980s and later integrated the WHO ICTRP Advisory Group). Several meetings were held in Europe starting in Brussels in 1991 to debate the importance of implementing registries and trial registration:

“A workshop on clinical trial registries, organized by Jean-Pierre Boissel (Lyon) and Kay Dickersin (Baltimore), was held in Brussels on July 12. The participants agreed on the need for all trials to be registered, and that there should be a directory of registries, both national and supranational.” [18].

In their 1993 meta-analysis study, Dickersin & Min demonstrated that “in every case, failure to publish was investigator-based, and not due to editorial decisions.” [13]; and acknowledged that “the identification of planned and ongoing trials will be a continuing challenge until registration is required of investigators (…). Who will take the lead?” [13].

The grounds for tackling publication bias and misreporting in clinical research —and specifically in pharmaceutical sponsored trials— were well documented and grounded. Following I. Chalmers’ claim that “adequate reporting of clinical trials is required for both scientific and ethical reasons” [19], inadequate reporting can be grouped in two categories, to differentiate two dimensions of the problem. While the first line of these arguments can be seen as focusing on the “clinical” aspect, this is, the fact that these studies are made with human participants who volunteer to enroll, the second can be analyzed from the “trial” piece, or in other words, a standardized way of creating scientific knowledge.

### Misreporting and publication bias as an unethical practice

Health research, including pharmaceutical sponsored drug trials, relies on the participation of human subjects. Unfortunately, it is no secret that the history of medical experimentation has not been exempt of unethical practices, both in the US and overseas [10], [20]. Notwithstanding several of these infamous experiments took place under totalitarian regimes during the second World War, others correspond to trials conducted in the era of megatrials —multicentric, often multi-country studies—, directly contributing to the decay in public opinion of the drug industry. As the model of industrialization became a standard model of business, the demand for larger samples and cheaper, faster recruiting boosted the offshoring of trials to developing countries with weaker regulations or ethical codes of health research, in a process that has been described as the globalization of clinical trials [21]. This process raised concerns regarding accountability of US based companies when conducting trials overseas, especially in poor populations, with fatal outcomes or questionable use of placebo. How to measure transparency in offshored trials? How to make drug companies accountable for reporting the totality of clinical trials conducted out of the US accurately, where FDA regulations are not legally binding? The issue of global clinical trials prompted a report from the National Bioethics Advisory Commission, titled “Ethical and Policy Issues in International Research Clinical Trials in Developing Countries”, released in 2001 [22]. Aiming to deliberate on the question: “Can a research design that could not be ethically implemented in the sponsoring, developed country be ethically justified in the country in which the research is conducted?”, it provided a series of recommendations intended to reduce the risk of exploiting vulnerable populations.

While the events described above imply an atmosphere of mistrust on industry practices, debates over publication bias highlighted the backfires that these practices had for enrolling voluntary participants whose trust could be irreversibly damaged. Moreover, publication bias and misreporting could undermine the very action of participating in a trial: “Failure to provide adequate, publicly available reports of the results of clinical trials does injustice to the patients who have participated in them.” [19]. This argument of betrayed trust was echoed by several authors and actors in the trial business. Advocates to end bias and selective reporting not only aimed to end unethical practices occurring during and after trials, but also emphasized the importance of reporting trial findings to avoid unnecessary suffering stemming from questionable protocols, futile replication and public spending on ineffective trials already conducted but unpublished, misinformation in decision makers and healthcare providers, and as a form of improving access to patients and families who might be benefited by experimental treatments [10], [14], [19], [23], [24].

### Misreporting and publication bias as scientific misconduct

“One of the most common ways to bias trials is to present only part of the data —the part that makes the product look good— and ignore the rest” [12]. Angell highlights that these “suppression of negative results”, or selective reporting, is a conscious commercial strategy used by the industry in order to maximize profit at the expense of data accuracy. Paxil’s “study 329” constitutes a case of selective reporting as the data regarding dangerous effects in children was purposefully disguised under a report of general effectiveness. Nevertheless, this was a widespread reporting practice.

The misconduct that Chalmers argued in 1990 resonated like a motto throughout the decade, as it underscored not only the fact that publications in medical journals were inaccurate because of the studies themselves, but because the bias between what was being published and what wasn’t was eventually affecting his own endeavors: the quality of systematic reviews and meta-analyses of the Cochrane collaboration (created in 1993 in the UK), which aims to provide the highest quality of systematized evidence to inform decisions. While publication bias was not an element included in systematic reviews methodology during the 1990s and early 2000, as early as 1986 this issue was pointed out by Simes in his seminal review [17], who demonstrated that the conclusions could vary dramatically depending on the sources included. Unfortunately, controlling bias in publication was out of the scope of reviewers, putting these scholars also at risk of scientific misconduct [15], [24]. And more importantly, as the Paxil case showed, the industry’s selective reporting practices was putting the population at risk of unsafe medications —and not only vulnerable populations in low- and middle-income countries-.

Furthermore, it also affected credibility of medical journals, and the role of journal editors, as they were accepting for publication manuscripts without certainty of the accuracy of the reported data [12], [19], or even transparent information about the authors —due to practices of ghostwriting common in industry-sponsored articles [23]—. Both evidence from the early 1900 and 2000 s analyzed by Dickersin showed that the practice of publication bias was strongly associated with either the investigator or the sponsor (especially when sponsored by the industry), rather than editorial decisions [13], [23]. Nonetheless, Horton and Smith, both editors of The Lancet and the British Medical Journal by the turn of the century, acknowledged that journal editors were also actors with “a part to play”:

“Editors also have a part to play. During peer review, editors increasingly find themselves requesting copies of the original trial protocol to check against the final submitted report. That “protocol culture” has led one of us to begin (and the other to plan) a protocol registration scheme. Editors are unwilling to fill their journals with promises of what might be, but they can publish these protocols on their web sites, *perhaps linking them to a central registry*.” (italics by me) [24].

### Answering the questions: the rationale for (unified) registration

As reviewed above, the problems associated with pharmaceuticals publication practices were numerous and serious during the first half of the 2000s. Both publication bias and selective reporting were not only difficult to track in an increasingly globalized industry, but their effects were threatening to undermine the very mission of the evidence-based medicine paradigm: on commenting the antidepressants’ crisis, Rennie wrote: “This storm over SSRIs is a good demonstration that no clinician can possibly practice evidence-based medicine if prevented from seeing the evidence.” [2].

Initiatives for trial registries have a history as old as the discussion, with different degrees of success and sustainability over time, and increasing endorsement by key agencies [15]. Indeed, by the time the WHO became an actor in trial registration, ambitious projects such as the FDA clinicaltrials.gov platform —launched in 2001 in the US—, the Cochrane Controlled Register of Trials (CENTRAL) launched in 1996, and the International Standard Randomized Controlled Trial Number (ISRCTN, launched in 2000), were already in force. However, registration initiatives were as scattered as the problem, and each database functioned with their own standard of registered data and had different scopes. Nevertheless, there was consensus between the authors about rationale regarding the role of registries and publication bias: for a registry to be effective in counteracting bias, it had to register trials at their inception, that is, before patients were recruited. This would avoid publication bias as it would be possible to detect those trials, ongoing, interrupted or completed, despite their later results, and despite their subsequent publication or not. Depending on the data required by the registry, it would be possible to avoid selective reporting by knowing beforehand details from the protocol and expected outcomes. With the massification of the internet, public status of trials would make it possible for patients, physicians, and other interested parties to identify trials they might be interested in participating or referring patients to, increasing transparency and ensuring access to the information in a timely manner [15], [24], [25].

One month before the Paxil lawsuit, The Lancet published a commentary by Timothy Evans, Metin Gülmezoglu and Tikki Pang, claiming that the WHO had an essential role in the task of trial registration, after a meeting held in London a few days earlier [25]. In this Commentary, Evans and colleagues provide a concise synthesis of the previous debate on the issue. Encompassing the major problems and arguments regarding transparency and registration, they underscore the potential of the WHO in the matter of registration, as “there is no registry that has comprehensive international coverage. There is a need for coordinated international collaboration to either build a single register or to link together all that exist.” [25]. Indeed, by 2004 the WHO was eager to step in the discussion.

## 2004–2006: The WHO picks up the gauntlet on trial registration

### The turning point: 2004

“It took a really disastrous year in 2004 for the regulators to be forced to account.” [26].

In Rennie’s words, 2004 represents the year when the idea of trial registration switched from “ignored” to “irresistible” [2]. As well, Jacky Law states that 2004 sets a tipping point for the relationship between the pharmaceutical industry and the regulators [26]. Indeed, on April of 2004, the WHO published a news release informing “that, from today, all randomized controlled trials approved by the WHO ethics review board will be assigned an International Standard Randomized Controlled Trial Number (ISRCTN)”. The release, titled “WHO leads drive for international coordination of clinical research” [27], mentions the problem of publication bias and how this measure intended to tackle it. However, there is no mention of the WHO projecting to have a registry platform of its own.

Two events from 2004 can be read as precipitating the involvement of the WHO in the plan of launching a proper platform. The first one is the Paxil lawsuit both in the US and the UK. The second corresponds to the statement from the International Committee of Medical Journal Editors (ICMJE), published in September. In this editorial, published simultaneously in the eleven journals members of the committee at the time, they stated: “The ICMJE member journals will require, as a condition of consideration for publication, registration in a public trials registry” [28]. The editorial also outlined that, although they did not advocate for any particular registry, the only one that met their requirements by then was clinicaltrials.gov. This last point was problematic, as the ICMJE released a second statement just a few weeks after the first, acknowledging that the clinicaltrials.gov platform only accepted US sponsored trials for registration at the time [29].

That same month, during a Cochrane Colloquium in Ottawa, the Ottawa Group was initiated by over a hundred experts in the subject, in an open meeting initiated by the Canadian Institutes of Health Research [30]. A year later, they published the first part of the Ottawa Statement in the British Medical Journal [31].

### From the Mexico Summit to the launch of the ICTRP

That same year in November, the WHO held the Mexico Ministerial Summit on Health Research, under the motto: “Knowledge for better health: strengthening health systems” [32]. With representatives from 58 countries, the official statement recognized that

“Research results must be published, documented in internationally accessible registers and archives, and synthesized through systematic reviews. These actions can help to inform decisions about support for new research and to build public confidence in science.” [32].

And specifically, called on:

“All major stakeholders, facilitated by WHO Secretariat, to establish a platform linking a network of international clinical trials registers to ensure a single point of access and the unambiguous identification of trials.” [32].

For the first time the WHO was acknowledging in an official document the necessity of a platform to facilitate access in a global scale to trial registries. This landmark is largely attributed to the statement of the ICMJE both by experts from the WHO [33], and Dickersin & Chalmers [23]. After the Mexico statement, things moved fast. The Statement was ratified at the 58th World Health Assembly held in May of 2005. In the WHA58.34 Resolution, the Assembly

“Called upon “the global scientific community, international partners, the private sector, civil society, and other relevant stakeholders, as appropriate… to establish a voluntary platform to link clinical trial registers in order to ensure a single point of access and the unambiguous identification of trials with a view to enhancing access to information by patients, families, patient groups and others.” [34].

Remarkably, the resolution wording alludes to the motive of “enhancing access” to patients and laypeople (families, patient groups). No mention to scientists or doctors, much less pharmaceutical industry stakeholders. It explicitly establishes the voluntary character of the platform. And as already outlined in Mexico Statement, the new platform is not intended to be a trial registry per se, but rather a portal — “a single point of access”— linking to trial registries.

According to the WHO Drug Information 2005 report, “in April 2005, a consultation was convened by WHO to initiate a framework for development of the international clinical trial registration platform.” [35]. The WHO ICTRP was established in August of 2005, and the platform was launched in May of 2006. The International Clinical Trial Registry Platform (ICTRP, http://www.who.int/ictrp), is embedded as a section of the WHO website, with its own repository of relevant publications and official documents. Prior to its launch, it was publicized by the WHO Bulletin: “WHO clinical trials initiative to protect the public” [36]. In the news release, it is stated that “The goal —of the ICTRP— is to increase transparency and accountability on the part of companies and institutions that do clinical research, and, in turn, boost public trust and confidence in that research.”. It also claims the suitability of the WHO: “WHO is best placed to do this as a global body representing 192 Member States that is able to set norms and standards in research, policy and practice.”. The release mentions the Paxil and Vioxx cases, and the public reaction of outrage. It also clarifies that the WHO has no intention of implementing a trial registry of its own.

Along with the platform, the WHO published the Standards for Clinical Trial Registries. The slogan of the platform and the Standards is “The registration of all interventional trials is a scientific, ethical and moral responsibility” [37]. The Standards were designed following the ICMJE requirements and they intend to identify the trial registries and databases suitable for being included in the platform, by establishing a 20-item dataset of information that trials must inform when registered. The Standards document establishes that:

“The mission of the ICTRP is to ensure that a complete view of research is accessible to all those involved in health-care decision-making. This will improve research transparency and will ultimately strengthen the validity and value of the scientific evidence base.” [37].

It also mentions that the ICTRP was created after the demand of “countries through the World Health Assembly”. This official document though, following the language of the WHA, does not mention the issue of publication bias or the role of pharmaceutical companies. Rather, the argument is again set in terms of improving access to people that may not be involved in research design or implementation, but in health decision-making. As registration is voluntary, the language is in terms of “responsibility”, avoiding negative wording like “scientific misconduct” (to echo Chalmers), “misreporting”, or even “bias”; in turn, it adds a “moral” dimension that is absent in the scientific debate that preceded the creation of the ICTRP.

This rationale has similarities and differences with the Ottawa Group Statement, which held that “Above all, international trial registration is necessary to fulfill ethical obligations to research participants” [31]. Although both acknowledge the centrality of ethics, the WHO places people “outside” trials as the main group benefited by transparency while the Ottawa Group centers the benefit in human participants “inside” the trial setting. Interestingly, the WHO claim of “ensuring complete view of research” via the platform, is technically inaccurate, as the registries were then mostly committed to tracking the existence of trials and some minimum dataset with their main characteristics, but not their results or other aspects of research.

A year later, in May 2007, the ICTRP launched the Clinical Trial Search Portal (CTSP), a tool for advanced navigation in the platform. By the same year, the ICMJE, after three years since their initial statement —with subsequent editorials and announcements regarding the progress and difficulties in the process of updating the registration of trials— claimed that the registration initiative had had significant progress: “Three years ago, trials registration was the exception; now it is the rule.” [38].

### The standpoint of the industry

What was the position of pharmaceutical companies regarding trial registration? In general terms, by the time the events described above happened, the industry was well organized and had strong mechanisms of public relations and lobbying. In the US, the Pharmaceutical Research and Manufacturers of America (PhRMA) group, founded in 1958, has been the major organism in charge of unifying the position of the industry and their influence. This influence has been exerted mainly through political lobbying. In 2003, the industry spent $108.6 million in federal lobbying in the US government, of which $16 million corresponds to PhRMA [39].

In terms of trial registration and findings disclosure, GlaxoSmithKline led the way. In 1999 —by then still Glaxo Wellcome— implemented a trial register for its trials in the company’s website, nonetheless it didn’t include information about study results [40]. A few years later, as a response to the creation of the All Trials Initiative in January of 2003, GSK was the first to sign up for the campaign [41]. However, it was highly criticized for what the company meant by “disclosure”: it set up an experts committee to determine unilaterally which patient level data could be accessed by the public. A year and a half later, the Paxil lawsuit was settled, with one of the measures to be taken by GSK being the prompt posting in its website of all the company trials and their results [42], which they did, becoming “the first company to launch an internet-based clinical study register” [43].

In general, the industry’s position oscillated between demonstrations of willingness to increase transparency and reluctance to registration and, specifically, of trial results disclosure. Dickersin & Rennie [15] summarized the resistance from the industry in 2003 as:

“Reasons for avoiding registration given to each of the authors in various public and private meetings over the years include protection of information about products under development, patents, and information about good recruitment centers, and not wanting to be bothered by dealing with consumers and others who contact them for information.” [15].

In short, the industry’s arguments combine a rationale of technical notions to explain the necessity of keeping trial information out of the public eye, though according to these experts, among others, the reasons ultimately were based on commercial threats. For Angell [12], FDA regulations in force at the time were also a source for perpetuation of both publication bias and selective reporting, as the agency had no control over publication practices. Not surprisingly, when Wager, Field & Grossman [44] published in 2003 their recommendations for good publication practices for pharmaceutical companies —which included a unique study identifier number—, they were not well received by the industry.

In January of 2005, the International Federation of Pharmaceutical Manufacturers & Associations (IFPMA), published the Joint Position on the Disclosure of Clinical Trial Information via Clinical Trial Registries and Databases. In this document, they distinguish between “registry” (for ongoing trials) and “database” (for completed trials results). This distinction is key to the industry, as results disclosure has historically been a source of more resistance than registration. Accordingly, the IFPMA established a deadline of 21 days to register a trial since initiation of patient enrolment, and for trial databases, “results should be posted no later than one year after the medicinal product is first approved and commercially available in any country.” [45]. As well, it establishes that “any one of a number of free, publicly accessible, internet-based registries should achieve the intended objectives”.

Nevertheless, the companies gave a sign of concrete resistance when it came the time to endorse the Ottawa Group Statement. In her article published on November of 2005, Krleža-Jerić [30] noted that not a single company endorsed the Statement (published in May), which she qualified as “unexpected”, and a “failure”. At the same time, she recognized that rather than an exception, this lack of endorsement was consistent with other practices by the industry, such as “fields left empty or filled in with meaningless information by certain pharmaceutical companies in these registries (such as clinicaltrials.gov)”.

Regarding the ICTRP, GSK issued a statement in January of 2006 [46] to justify the need for delayed disclosure on specific situations. They claimed that early disclosure could harm some innovative initiatives as third parties might “copy” (with quotation marks in original) these innovative ideas, undermining competitiveness. They also called for the WHO to focus on “results registries and databases as it is the results of trials that can impact patient care.”, rather than prospective trial registration. Finally, they exhorted the WHO to clarify which trials are supposed to be registered, as it remained unclear from the official statements.

Two months later PhRMA sent a strong statement during the second round of Formal Consultation on Disclosure Timing comments of the ICTRP, mentioning its ongoing commitment to transparency and registration of all trials, except exploratory ones [47]. More importantly, it claims that “Registration of a clinical trial alone does not fully meet the full transparency objective. This WHO focus on registration and the associated data elements misses this important connection.”. Finally, it urges the WHO to “consider the resource implications of trial transparency on all parties and not just the pharmaceutical industry.”. In a commentary published by The Lancet in May, Ida Sim —by then the Project Manager of the ICTRP— and colleagues, commenting on the consultation, remarked: “the arguments for delayed disclosure were neither convincing nor compelling.” [48].

## Discussion

The economic and geographic expansion of pharmaceutical research has raised significant concerns regarding transparency both in publishing practices and ethical treatment of trial participants. Since the mid-1980s scientists expressed their worries about the widespread practices of publication bias and selective reporting of results of industry-sponsored trials, which ultimately affected the quality of evidence and concealed valuable information concerning effectiveness, drug safety and mismanagement of vulnerable populations enrolled in off-shored trials [18], [19], [23].

Transparency concerns have affected credibility and public trust in the drug industry and their management of data, moreover after public scandals concerning unethical practices with undeserved groups —like the AZT trials in Africa— and concealment of results —especially the Paxil and Vioxx (rofecoxib) scandals, among others— [2], [10], [12]. To avoid these research malpractices and restore transparency, since the early 2000s several clinical trial public registries have been launched. However, none of these registries had a global scope. In 2004, the WHO entered the debate for trial registration launching in 2006, the International Clinical Trial Registry Platform (ICTRP). The platform consists of an online, public, free access portal that contains the links to official clinical trials registries recognized by the WHO.

As a technocratic apparatus, the ICTRP performs a surveillance role of the pharmaceutical sector. Indeed, it is impracticable for the WHO (or any other global public actor, if any) to perform real-world audits of all clinical trials being conducted at a given time. In this sense, the WHO enacts a discourse of ethicality where transparency becomes a matter of representation.

Under the form of dataveillance —the surveillance of data instead of subjects, or in other words, subjects as data— [49], an online portal makes transparency not only reachable, but quantifiable. Exposing the trial to public scrutiny it is equated to righteous behavior, i.e. “good research practices”, and thus, held as a proof that the trial is being conducted accordingly, and the sponsor is worth of public trust. This allows the WHO to exercise leadership —and thus, power— in the global health arena, and to have a voice in the public debate of how neoliberal capitalism shapes drug development and clinical research [50]. As well, the WHO stands as the guardian of evidence-based medicine, the rising paradigm for decision making in medicine and public health. Indeed, it was not a coincidence that eminent early experts of evidence-based healthcare were the most vocal advocates for the WHO stepping in.

The history of trial registration has continued to unfold since the ICTRP launch. More actions and debates have followed. Two are worth mentioning: the first is the passing of the FDA Amendment Act into law, in 2007. The FDAAA establishes more explicitly the requirements for trial registration and disclosure of results, with monetary penalties for delays committed by trials federally funded [50]. The second is the inclusion of trial registration in the 2008 update of the Helsinki Declaration [32]. However, even though the importance of trial registration has been endorsed by key agencies in the field of medical research and bioethics, a decade after its launch showed that compliance with registration is far from optimal [15], [33], [51].

Considering that trial registration is only a step towards medical and pharmaceutical research transparency, important questions arise in terms of the feasibility of holding the pharmaceutical industry accountable for transparency. In this scenario, critical scientific appraisal of the issue through historical textual analysis such as this, is a valuable input to enrich the discussion around the WHO past and present role in global health, as it has been analyzed in other fields in this same journal [52]. Limitations of the present article can be observed in terms of its reproducibility as understood in the health sciences, as the method utilized did not attempt to follow a “synthesis of evidence” methodology. In this sense, this paper does not intend to establish an objective truth on the issue described, but to offer a new perspective on the facts analyzed throughout the article.

## Conclusion

The problem of trial registration is worrying not only for the human subjects that have participated and continue to enroll in clinical trials, but also because the consequences of publication bias and selective reporting are simply impossible to size. Unsafe medications that have been approved to be on the market put everyone at risk, including present and future patients.

In its historical context, the creation of the ICTRP and the positions from the different parties involved, can be read as a process where the WHO intended to reaffirm its presence as a vital global health stakeholder; not as an economic actor (vastly exceeded and outnumbered by the big pharma revenues), but as an actor committed with values like transparency and responsibility rather than tangible goods.

In this scenario, it is striking that the WHO delayed almost two decades to act since Simes showed evidence of the impact of the industry’s publishing practices in science. The fact that compliance with trial registration is far from optimal raises the question if the WHO action was already too late. Furthermore, it raises the question about the real power of the WHO over private drug development. The prominent absence of openly mentioning the practices of bias and misreporting in some of the major WHO official documents in the matter is concerning. Why did the WHO avoid to publicly address the scientific misconduct in some of its main communications?

The history of trial registration, and why it was (and is) needed, shows the difficulty of trying to regulate a health enterprise that has transformed into a global business. Moreover, it shows the challenges of globalization and how easier (and faster) it is to globalize business compared to good practices or, in other words, how feasible it was for the industry to avoid transparency. As well, it does raise the question of why it has been so hard to undo these trends. Indeed, the history of the movement for trial registration is not a history of regulation success, or at least not yet.

## Data Availability

All sources cited are publicly available where indicated.

## Abbreviations

WHO: World Health Organization
ICTRP: International Clinical Trials Registry Platform
R&D: Research and Development
ICMJE: International Committee of Medical Journal Editors
FDA: Food and Drug Administration
CENTRAL: Cochrane Controlled Register of Trials
ISRCTN: International Standard Randomized Controlled Trial Number
PhRMA: Pharmaceutical Research and Manufacturers of America
IFPMA: International Federation of Pharmaceutical Manufacturers & Associations
FDAAA: Food and Drug Administration Amendments Act
